# The impact of adenomyosis on IVF outcomes: a prospective cohort study

**DOI:** 10.1093/hropen/hoab015

**Published:** 2021-04-19

**Authors:** Chloe Higgins, Hugo Fernandes, Fabricio Da Silva Costa, Wellington P Martins, Beverley Vollenhoven, Martin Healey

**Affiliations:** 1 Women’s and Newborn Programme, Monash Health, Clayton, VIC, Australia; 2 Newlife IVF, Box Hill, VIC, Australia; 3 Royal Women’s Hospital, Parkville, VIC, Australia; 4 Department Obstetrics & Gynaecology, Monash University, Clayton, VIC, Australia; 5 Department of Gynecology and Obstetrics, Ribeirão Preto Medical School, University of São Paulo, São Paulo, Brazil; 6 Department of Reproductive Medicine, SEMEAR fertilidade, Ribeirão Preto, São Paulo, Brazil; 7 Monash IVF, Clayton, VIC, Australia; 8 Department Obstetrics & Gynaecology, University of Melbourne, Parkville, VIC, Australia

**Keywords:** adenomyosis, IVF, fertility, female infertility, embryo transfer

## Abstract

**STUDY QUESTION:**

Does the presence of adenomyosis in women treated with IVF alter IVF outcomes?

**SUMMARY ANSWER:**

Adenomyosis does not significantly alter IVF outcomes when adjusted for confounding factors including maternal age and smoking status.

**WHAT IS KNOWN ALREADY:**

Studies evaluating adenomyosis and its impact on infertility, particularly when focusing on IVF, remain controversial. Many studies report that adenomyosis has a detrimental effect on IVF outcomes, however age is strongly related with both the prevalence of adenomyosis and worse reproductive outcomes.

**STUDY DESIGN, SIZE, DURATION:**

A prospective cohort study of women undergoing 4002 IVF cycles who had undergone a screening ultrasound assessing features of adenomyosis from 1 January 2016 to 31 March 2018 at a multi-site private fertility clinic. Of these women, 1228 fulfilled the inclusion criteria and commenced an IVF cycle, with a subset of 715 women undergoing an embryo transfer (ET). Women were defined as having adenomyosis if there was sonographic evidence of adenomyosis on ultrasound as per the Morphological Uterus Sonographic Assessment criteria, and were then compared to women without.

**PARTICIPANTS/MATERIALS, SETTING, METHODS:**

All women at a private multi-site IVF clinic who underwent a standardised ultrasound to identify features of adenomyosis and also commenced an IVF cycle were assessed for their outcomes. These included clinical pregnancy (defined as the presence of a gestational sac on ultrasound at 7 weeks’ gestation), clinical pregnancy loss, number of cancelled cycles, number of useful embryos for transfer or freezing and live birth rates. As a secondary aim, initiated stimulation cycles and those that had an ET were analysed separately to determine when an effect of adenomyosis on IVF might occur: during stimulation or transfer.

**MAIN RESULTS AND THE ROLE OF CHANCE:**

When adjusting for confounders, women with and without sonographic features of adenomyosis had no significant differences in most of their IVF outcomes including live birth rates.

**LIMITATIONS, REASONS FOR CAUTION:**

Adenomyosis had a detrimental impact on IVF outcomes prior to adjusting for confounding factors. No allowance was made for the possibility that confounding factors may merely reduce the effect size of adenomyosis on IVF outcomes. Second, despite a power calculation, the study was underpowered as not all fresh cycles led to an ET.

**WIDER IMPLICATIONS OF THE FINDINGS:**

This is one of the largest studies to evaluate adenomyosis and IVF outcomes, while also importantly adjusting for confounding factors. The results suggest that adenomyosis does not have the detrimental impact on IVF that has previously been suggested, possibly reducing the importance of screening for and treating this entity.

**STUDY FUNDING/COMPETING INTEREST(S):**

The study received no external funding. The authors declare no conflicts of interest.

**TRIAL REGISTRATION NUMBER:**

ACTRN12617000796381.

**WHAT DOES THIS MEAN FOR PATIENTS?**

Adenomyosis is a poorly understood medical condition in which the lining of the uterus grows inside the muscle of the uterus. It is not clear whether this affects the chance of pregnancy, but this is especially important to know when doing IVF treatments for infertility. Most research in this area is made up of small studies that contradict each other. This study used ultrasounds to diagnose adenomyosis and looked at large groups of women to try to detect more accurately whether having adenomyosis reduces the success of IVF treatments. The researchers looked at women with and without adenomyosis having IVF and found no difference in the chances of giving birth to a live baby. This suggests that the diagnosis and treatment of adenomyosis may not be as important to IVF treatments as previously thought. The researchers hope this can start to shed some light on adenomyosis and encourage more research to be done in this area to improve our understanding of this condition.

## Introduction

Adenomyosis is a benign invasion of endometrial glands and stroma into the uterine myometrium (Bergeron *et al.*, 2006; Campo *et al.*, 2012; Van den Bosch *et al.*, 2015). It can present with heavy menstrual bleeding, dysmenorrhoea, abnormal bleeding or be asymptomatic (Bergeron *et al.*, 2006; Meredith *et al.*, 2009; Champaneria *et al.*, 2010).

While there are no internationally agreed diagnostic criteria for adenomyosis on either ultrasound or MRI (Kunz *et al.*, 2005; Bergeron *et al.*, 2006; Benagiano *et al.*, 2009; Wang *et al.*, 2009; Maheshwari *et al.*, 2012; Chapron *et al.*, 2020), the Morphological Uterus Sonographic Assessment (MUSA) consensus statement lists several important ultrasound features for diagnosing adenomyosis (Van den Bosch *et al.*, 2015). These include anteroposterior asymmetry of the myometrium, ill-defined lesions, fan-shaped shadowing, myometrial cysts, hyperechoic islands, echogenic subendometrial lines and buds, translesional vascularity and an irregular or interrupted junctional zone (Van den Bosch *et al.*, 2015). Collectively, these features are often referred to as sonographic evidence of adenomyosis (SEOA).

Adenomyosis is suggested to affect 8–27% of the population (Maheshwari *et al.*, 2012; Naftalin *et al.*, 2012); however, this incidence reportedly increases to 50–85% in women with infertility (de Souza *et al.*, 1995; Zangos *et al.*, 2004; Benagiano *et al.*, 2009). Despite this, a link between adenomyosis and infertility is contentious owing to small study sizes and possible confounders, such as age, the presence of endometriosis and a higher rate of pelvic imaging, in women undergoing workup for infertility compared with their unaffected counterparts (de Souza *et al.*, 1995; Benagiano *et al.*, 2009; Benaglia *et al.*, 2014; Morassutto *et al.*, 2016; Yu *et al.*, 2020).

There is limited published data available on SEOA in the setting IVF. Several studies suggest that it is associated with poorer outcomes (Chiang *et al.*, 1999; Maubon *et al.*, 2010; Mijatovic *et al.*, 2010; Youm *et al.*, 2011; Maheshwari *et al.*, 2012; Salim *et al.*, 2012; Mavrelos *et al.*, 2017; Buggio *et al.*, 2018; Stanekova *et al.*, 2018; Sharma *et al.*, 2019). While most studies have not formally controlled for maternal age (de Souza *et al.*, 1995; Chiang *et al.*, 1999; Kissler *et al.*, 2007; Youm *et al.*, 2011; Naftalin *et al.*, 2012; Salim *et al.*, 2012), others have and still report poorer IVF outcomes in patients with SEOA (Maubon *et al.*, 2010; Thalluri and Tremellen, 2012; Mavrelos *et al.*, 2017; Stanekova *et al.*, 2018). Contradictory studies also exist, showing no differences, when controlling for age, in rates of pregnancy, implantation, miscarriage and live birth for patients having IVF treatment who do or do not have SEOA (Camargo *et al.*, 2001; Costello *et al.*, 2011; Yan *et al.*, 2014).

A call has, therefore, been made for large studies matched for confounding factors to clarify this contentious topic (Chiang *et al.*, 1999; Salim *et al.*, 2012; Thalluri and Tremellen, 2012; Yan *et al.*, 2014; Dueholm, 2017).

The aim of this study was to perform a prospective cohort study to assess whether the presence of SOEA affected live birth rate following a fresh or frozen embryo transfer (ET) and to identify where in the reproductive process such an effect may occur.

## Materials and methods

### Study population and design

This is a prospective cohort study involving a private multi-site IVF clinic with a co-existing ultrasound service. Data were entered prospectively in a standardised manner into the clinic database to avoid issues of recall bias.

All women aged 18–45 years who underwent an ultrasound between 1 January 2016 and 31 March 2018 were assessed for SEOA as part of a standardised ultrasound evaluation. Those women who then had an episode of fertility treatment (stimulated IVF or ET cycles) during this same time were included in the study.

**Figure 1.**
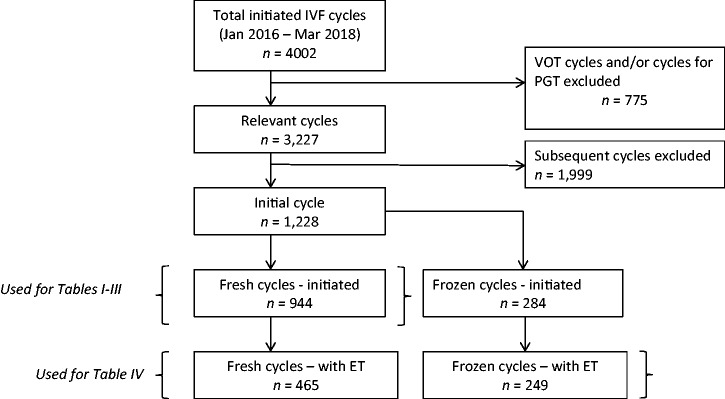
**Flow chart of study IVF cycles in a prospective analysis of the impact of adenomyosis on IVF outcomes.** PGT, preimplantation genetic testing; ET, embryo transfer; VOT, vitrification oocyte thaw cycles

We identified 4002 episodes of fertility treatment (cycles), of which 775 were excluded for being vitrification oocyte thaw (VOT) cycles or for preimplantation genetic testing (PGT). The remaining 3227 cycles were then limited to one cycle per participant—the initial stimulated IVF or frozen ET cycle during the study period (n* *=* *1228 cycles). Fig. 1 documents the exclusion process. A total of 944 stimulated IVF cycles and 284 frozen ET cycles were initiated. In 714 of these, a fresh or frozen embryo was transferred.

### IVF cycles

IVF cycles were undertaken using previously described protocols (Motteram *et al.*, 2015; Higgins *et al.*, 2018).

### Data sources and measurement

Data were extracted from the standardised databases maintained by the IVF clinic and the ultrasound service.

Sonographers with extensive training and experience in gynaecological ultrasound performed the patient ultrasounds. They were trained specifically to report signs of adenomyosis as per the MUSA criteria. All scans were then reviewed by three senior specialists with Certification in Obstetrical and Gynaecological Ultrasound. Scans were reviewed using 2- and 3-dimensional views as well as videoclips for all patients. SEOA was defined by the presence of any one or more features as defined by MUSA criteria including myometrial cysts, loss of endo-myometrial interface, venetian blind shadowing, diffuse coarse echogenicities (also known as echogenic buds or myometrial echogenic islands), increased vascularity or increased antero-posterior myometrial diameter. Severe SEOA was defined as three or more positive markers of SEOA on ultrasound; however, data on location and extent of adenomyosis were not extracted. Evaluations were performed using GE, E10 and E8 ultrasound machines using 3-dimensional imaging of the uterus in all cases.

### Outcome measures and statistical analysis

The primary outcome measures were clinical pregnancy and live birth rate. Secondary outcomes included number of cycles cancelled, number of follicles at trigger, total number of eggs collected, total number of eggs fertilised, fertilisation rate, total number of ‘useful embryos’ (embryos able to be transferred or frozen), cycles with no useful embryos and pregnancy loss rates. Clinical pregnancy was defined as the presence of a gestational sac on ultrasound at 7 weeks gestation and live birth was defined as the birth of a live infant at greater than 20 weeks gestation.

Of all data collected, 3.9% data points were missing. Multiple imputation was performed using ‘mi’ in STATA (version 15) with 100 imputations. All regression analyses were performed on the imputed data using ‘mi estimate’, and pooled results are presented.

Comparisons of demographic and cycle variables between women with and without SEOA were performed using univariate logistic regression.

All outcomes were initially modelled using univariate regression with SEOA status as the independent variable. Adjusting was then performed with explanatory variables. For cancelled cycles and follicle count outcomes, these were the following: patient age (centred on the mean and including age squared, as the relationship with age is non-linear and better modelled with a quadratic function), BMI, smoking status, infertility aetiologies, nulliparity status, number of previous ART cycles, total FSH dose, maximum oestradiol concentration and cycle stimulation type. Follicle count was also added for the outcomes of number of eggs collected, number of fertilised eggs and fertilisation rate. For analysis of the number of useful embryo and cycles with no useful embryos, the total number fertilised eggs was included in the model. The number of useful embryos was included in modelling for clinical pregnancy, pregnancy loss and live birth rates. Initiated stimulation cycles and those that had an ET were analysed separately to determine when the effects of adenomyosis on IVF might occur: during stimulation or transfer. For binary outcomes, logistic regression was used; for count outcomes, negative binomial regression; for ordered category outcomes, ordered logistic regression; and for continuous variables, linear regression.

Using initial data and adjusted models, rates divided on SEOA status were calculated and predicted for all initiated cycle outcomes: cancellation, no eggs were obtained, all embryos were frozen, no embryo to transfer, no pregnancy from a transfer, pregnancy loss and live birth. The adjusted logistic regression models included as independent variables: adenomyosis status, aetiology status (for endometriosis, polycystic ovaries, polycystic ovary syndrome, ovulation defect), patient age, smoking status, nulliparity status and treatment cycle number.

Sub-analysis was performed comparing severe SEOA and no SEOA. Analysis was also performed limiting to subjects’ first IVF stimulation cycle and then a separate analysis limiting to nulliparous subjects.

Findings were considered significant if *P *<* *0.05. Statistical analysis was performed using SPSS version 25 (IBM, Armonk, NY, USA), STATA version 15 (College Station, TX, USA) and Epi-Info version 3.5.3, (Centres for Disease Control and Prevention, Atlanta, GA, USA).

### Sample size calculation

The clinical pregnancy and live birth rate for an IVF cycle with a Day 5 fresh transfer was estimated to be 30% and 25%, respectively, from the IVF clinic’s previous audits. A 10% fall in clinical pregnancy or live birth rate was considered to be clinically significant. The ratio of patients with and without adenomyosis was estimated as 1:3. A sample size of 880 was calculated (power 80% and alpha 0.05) to be required, consisting of 220 patients with adenomyosis and 660 patients without (Stata version14) based on clinical pregnancy, with a sample size for live birth of 640 (160 with adenomyosis and 480 without).

### Ethical approval

This study was approved by the local ethics committee (MH 15172 M), and was registered midway through the study recruitment phase (May 2017) with the Australian & New Zealand Clinical Trials Registry (ACTRN12617000796381).

## Results

The markers of SEOA identified in this population and their prevalence are described in Table I. The most common finding in women with SEOA was diffuse coarse echogenicities on ultrasound (23%).

**Table I:** Prevalence of sonographic evidence of adenomyosis.

Marker	% (n)
Loss of Endometrial-Myometrial interface	11 (100)
Venetian blind shadowing	14 (135)
Increased vascularity	10 (97)
Increased anteroposterior diameter	13 (119)
Diffuse coarse echogenicities	23 (217)
Myometrial cysts	8 (73)

**Number of SEOA markers present**	

0	68 (643)
1	14 (135)
2	4 (34)
3	4 (40)
4	6 (52)
5	3 (30)
6	1 (10)

N = 944.

SEOA, sonographic evidence of adenomyosis.

The characteristics of patients commencing a stimulation cycle with and without SEOA are illustrated in Table II. Of note, age, smoking status and total FSH dose were significantly different between women with and without SEOA.

**Table II:** Patient demographics by presence of SEOA.

	With SEOA (N = 301)	Without SEOA (N = 643)	*P*
Age at OPU (years)	37.4 (0.3)	36.0 (0.2)	**<0.01**
Smoker	6.3% (19/301)	2.2% (14/643)	**<0.01**
BMI (kg/m^2^)	26.2 (0.3)	25.8 (0.2)	0.42
Missing	44	84	
Parity:			0.22
0	93.0% (280/301)	93.9% (604/643)	
≥1	7.0% (21/301)	6.1% (39/643)	
Subfertility Aetiology:			
PCO	3.5% (11/301)	6.7% (43/643)	0.07
PCOS	6.6% (20/301)	4.0 (26/643)	0.09
Ovulation defect	6.3% (19/301)	6.1% (39/643)	0.88
Ovarian failure	1.3% (4/301)	1.4% (9/643)	0.93
Poor responder	0.3% (1/301)	1.2% (8/643)	0.21
Endometriosis	11.3% (34/301)	7.8% (50/643)	0.08
Endometrioma	0.3% (1/301)	0.0% (0/643)	—
Fibroids	1.0% (3/301)	1.2% (8/643)	0.74
Genetic	0.0% (0/301)	0.8% (5/643)	—
Cancer	0.0% (0/301)	0.9% (6/643)	—
Tubal factor	10.6% (32/301)	8.4% (54/643)	0.27
Male factor	16.3% (49/301)	14.5% (93/643)	0.47
Idiopathic	46.2% (139/301)	46.3% (298/643)	0.96
Other	14.3% (43/301)	11.5% (74/643)	0.23
Not documented	61.5% (185/301)	67.0% (431/643)	0.09
Number of previous OPU:			0.09
0	82.0% (201/245)	86.4% (483/559)	
1	9.0% (22/245)	6.6% (37/559)	
2	3.3% (8/245)	3.6% (21/559)	
>2	5.7% (14/245)	3.2% (18/559)	
Missing data	56	84	
Number of previous ET:			0.07
0	83.7% (205/245)	88.0% (492/559)	
1	6.9% (17/245)	4.7% (26/559)	
2	3.7% (9/245)	2.0% (11/559)	
>2	5.7% (14/245)	5.4% (30/559)	
Missing data	56	84	
Stimulation type:			0.23
Agonist	5.4% (14/257)	3.5% (20/571)	
Antagonist	89.9% (231/257)	91.9% (525/571)	
Other	4.7% (12/257)	4.6% (26/571)	
Missing data	44	72	
Total FSH dose (IU/L)	3068.6 (173.6)	2677.5 (61.0)	**0.02**
Max oestradiol level (pmol/L)	4833.6 (262.8)	5428.8 (187.1)	0.07
Insemination type:			0.15
Conventional	18.4% (45/244)	23.6% (131/556)	
Half & half	4.9% (12/244)	5.8% (32/556)	
ICSI	76.7% (187/244)	70.7% (393/556)	
Cancelled cycles	57	87	

OPU, oocyte pick up; ET, embryo transfer; PCO, polycystic ovaries; PCOS, polycystic ovary syndrome.

Data presented as mean (SD) or % (n).

Differences in cycle characteristics between patients with and without SEOA are explored in Table III. Cancelled cycle rate was higher, while the number of eggs collected, number of eggs fertilised and total number of useful embryos were all significantly lower in women with SEOA on univariate analysis. These findings were no longer significant when analyses were adjusted for patient demographics and stimulation factors.

**Table III:** Cycle characteristics and outcomes by presence of SEOA.

	With SEOA	Without SEOA	Univariate *P*	Adjusted *P*
**Stimulation & collection results:**	**N = 301**	**N = 643**		
Cancelled cycles	18.9% (57/301)	13.5% (87/643)	**0.03**	0.51
Number follicles >11mm	9.7 (0.6)	10.4 (0.3)	0.20	0.24
Number of eggs collected	8.2 (0.5)	9.8 (0.3)	**0.01**	0.41
Number of eggs fertilised	3.9 (0.3)	4.9 (0.2)	**<0.01**	0.17
Proportion eggs fertilised	59.7% (1.9)	61.4% (1.2)	0.44	0.72
**Fresh cycle embryo results:**	**(N = 244)**	**(N = 556)**		
Number of Useful Embryos (ie: Transferred or Frozen embryos)	2.1 (0.1)	2.7 (0.1)	**<0.01**	0.52
Fresh cycles with zero useful embryos	21.7% (53/244)	15.1% (84/556)	**0.02**	0.10
N embryos transferred per cycle			0.23	0.25
0	46.7% (114/244)	39.8% (221/556)		
1	48.4% (118/244)	55.0% (306/556)		
2	4.9% (12/244)	5.2% (29/556)		
Embryo age at transfer			0.58	0.10
D2	0.0% (0/130)	1.2% (4/335)		
D3	37.7% (49/130)	39.4% (132/335)		
D4	1.5% (2/130)	0.6% (2/335)		
D5	60.8% (79/130)	58.8% (197/335)		
Embryo grade			0.79	0.58
A	29.2% (38/130)	26.3% (88/335)		
B	34.6% (45/130)	43.0% (144/335)		
C	28.5% (37/130)	23.0% (77/335)		
D	7.7% (10/130)	7.8% (26/335)		

Data presented as mean (SD) or % (n).

The fates of all stimulation cycles commenced are summarised in Table IV. Women with SEOA had a higher rate of cycle cancellation and there being no embryo for transfer. The clinical pregnancy rate after adjusting for confounding factors was significantly reduced in women with SEOA, while the lower live birth rate was not significantly different. Subgroup analysis performed between severe SEOA and no SEOA showed no significant difference in clinical pregnancy or live birth rates when adjusted for confounders, regardless of whether the initiated stimulation cycle or cycle with a fresh embryo was investigated (Supplementary Table SI).

**Table IV:** Clinical outcomes of initiated stimulation cycles by presence of SEOA.

Result of Cycle	With SEOA (N = 301)	Without SEOA (N = 643)	Crude OR	*P*	Adjusteda OR	*P*
Cycle cancelled	18.9% (57)	13.5% (87)	1.5 (1.0–2.2)	**0.03**	1.0 (0.7–1.6)	0.87
No eggs collected	0.7% (2)	1.1% (7)	0.6 (0.1–2.9)	0.54	0.4 (0.1–2.1)	0.28
No embryo transferred	56.8% (171)	47.9%% (308)	1.4 (1.1–1.9)	**0.01**	1.3 (1.0–1.8)	0.06
Freeze-all embryos cycle	19.6% (59)	20.2% (130)	1.0 (0.7–1.4)	0.83	1.2 (0.8–1.7)	0.35
No clinical pregnancy from ET	33.2% (100)	34.8% (224)	0.9 (0.7–1.2)	0.63	1.0 (0.7–1.3)	0.79
Clinical pregnancy	10.0% (30)	17.3% (111)	0.5 (0.4–0.8)	**<0.01**	0.6 (0.4–1.0)	**0.03**
Clinical pregnancy loss	5.0% (15)	8.4% (54)	0.6 (0.3–1.0)	0.06	0.6 (0.3–1.1)	0.12
Live birth	5.0% (15)	8.9% (57)	0.5 (0.3–1.0)	**0.04**	0.6 (0.3–1.2)	0.15

^a^ Logistic Regression prediction model included explanatory variables: age, smoking status, treatment cycle, aetiology status (endometriosis, ovulation defect, polycystic ovaries, polycystic ovarian syndrome), parity status. This analysis used pooled results from multiple imputation data. OR: odds ratio.

Data presented as % (n).

Table V summarises the outcomes limited to cycles where an ET occurred (either fresh or frozen) in women with and without SEOA. Women having a fresh ET cycle with SEOA had lower rates of clinical pregnancy compared with women without SEOA on univariate analysis. However, these differences were no longer statistically significant when adjusting for all explanatory variables.

**Table V:** Comparison of outcomes of fresh and frozen embryo transfers by presence of SEOA. Data presented as mean (SD) or % (n).

	With SEOA	Without SEOA	Crude OR	*P*	Adjusteda OR	*P*
**Fresh**	**N = 130**	**N = 335**				
Age at oocyte pick up	36.5 (5.0)	35.6 (4.1)		0.07		
Clinical Pregnancy	23.1% (30/130)	33.1% (111/335)	0.6 (0.4–1.0)	**0.04**	0.7 (0.4–1.1)	0.10
Clinical Pregnancy Loss	11.5% (15/130)	16.1% (54/335)	0.7 (0.4–1.3)	0.21	0.6 (0.3–1.3)	0.20
Live Birth	11.5% (15/130)	17.0% (57/335)	0.6 (0.4–1.2)	0.15	0.7 (0.4–1.4)	0.32
**Frozen**	**N = 79**	**N = 170**				
Age at oocyte pick up	37.1 (4.2)	35.9 (4.2)		**0.047**		
Clinical Pregnancy	40.5% (32/79)	40.0% (68/170)	1.0 (0.6–1.7)	0.94	1.2 (0.6–2.1)	0.62
Clinical Pregnancy Loss	16.5% (13/79)	16.5% (28/170)	1.0 (0.5–2.1)	1.00	0.9 (0.4–2.1)	0.88
Live Birth	24.1% (19/79)	23.5% (40/170)	1.0 (0.6–1.9)	0.93	1.3 (0.7–2.5)	0.48

^a^ Logistic Regression model included explanatory variables: age, smoking status, treatment cycle, aetiology status (endometriosis, ovulation defect, PCO, PCOS), BMI, parity status. This analysis used pooled results from multiple imputation data.

When limiting the analysis to the subject’s first IVF cycle, rather than the initial cycle, patients with severe SEOA had statistically significant reduced clinical pregnancy rates, but no difference in live birth rates following adjustments for confounders (Supplementary Table SII). A similar finding was also seen when limiting the analysis to nulliparous subjects (Supplementary Table SIII).

## Discussion

There is no clear consensus as to the impact or effects of SEOA on fertility (Maheshwari *et al.*, 2012) owing to limited published data available on SEOA in the setting of IVF. Several studies suggest that SEOA is associated with poorer outcomes (Mijatovic *et al.*, 2010; Maheshwari *et al.*, 2012) including significantly higher rates of spontaneous abortion (Chiang *et al.*, 1999), lower rates of implantation, clinical pregnancy and live birth, as well as generally poorer obstetric outcomes (Maubon *et al.*, 2010; Youm *et al.*, 2011; Salim *et al.*, 2012; Mavrelos *et al.*, 2017; Buggio *et al.*, 2018; Stanekova *et al.*, 2018; Sharma *et al.*, 2019). The number of features of SEOA identified also appears proportional to the severity of outcomes (Youm *et al.*, 2011; Mavrelos *et al.*, 2017). While most studies have not formally controlled for maternal age (de Souza *et al.*, 1995; Chiang *et al.*, 1999; Kissler *et al.*, 2007; Youm *et al.*, 2011; Naftalin *et al.*, 2012; Salim *et al.*, 2012), others have and still report poorer IVF outcomes in patients with SEOA (Maubon *et al.*, 2010; Thalluri and Tremellen, 2012; Mavrelos *et al.*, 2017; Stanekova *et al.*, 2018). Several retrospective studies and case reports have found an improvement in fertility when adenomyosis has been managed with GnRH agonist or levonorgestrel intrauterine hormonal treatments, surgical resection of adenomyomas, uterine artery embolization or magnetic resonance-guided focused ultrasound (Honore *et al.*, 1988; Silva *et al.*, 1994; Wang *et al.*, 2000; Wang *et al.*, 2006; Tremellen and Russell, 2011 Park *et al.*, 2016;; Liang *et al.*, 2019; Stanekova *et al.*, 2018). While this may suggest a possible causal relationship, further research is required (Tremellen and Russell, 2011).

Contradictory studies also exist, showing no differences, when controlling for age, in rates of pregnancy, implantation, miscarriage and live birth for patients having IVF treatment who do or do not have SEOA (Camargo *et al.*, 2001; Costello *et al.*, 2011; Yan *et al.*, 2014). A possible trend towards worse outcomes has been noted (Yan *et al.*, 2014) but the small sample size of these studies is a significant limitation.

A meta-analysis performed by Vercellini *et al.* concluded that while adenomyosis appeared to negatively impact IVF outcomes, larger studies were needed to confirm this effect (Vercellini *et al.*, 2014). Following this review, the same group published a contradictory case–control study that demonstrated asymptomatic adenomyosis did not adversely affect embryo implantation (Benaglia *et al.*, 2014). The most recent meta-analysis by Younes *et al.* concluded that adenomyosis was associated with reduced fertility and poorer pregnancy outcomes in IVF. These outcomes improved following treatment of adenomyosis; however, the analysis was limited by the small size, the variable quality of the studies and the lack of adjusting for confounding factors (Younes and Tulandi, 2017). While the literature is divided most studies, including two meta-analyses, suggest that SEOA is associated with worse IVF outcomes (Vercellini *et al.*, 2014; Younes and Tulandi, 2017).

Our study primarily sought to assess the effect of SEOA on clinical pregnancy and live birth rates. Contrary to the literature, we have shown that SEOA has no statistically significant effect on live birth. This finding was seen when we assessed fresh IVF stimulation cycles but also when we looked purely at the transfer of fresh or frozen embryos. Although the presence of SEOA appeared to have a significant impact on clinical pregnancy rates, it did not significantly impact on other outcome measures in the IVF process from follicle number through to clinical pregnancy loss and live birth.

In the current study, several outcome measures on univariate analysis were significantly altered in patients with SEOA. These differences were reduced and no longer significant following adjusting for important confounding factors such as age and smoking. The mixed findings in the literature may, at least in part, be explained by whether or not investigators adjusted outcomes for explanatory factors such as age (Chiang *et al.*, 1999; Salim *et al.*, 2012; Thalluri and Tremellen, 2012; Yan *et al.*, 2014; Dueholm, 2017).

Increasing age is associated with lower ovarian reserve and quality as well as higher rates of aneuploidy. This is slightly reduced in frozen transfers as only high-quality embryos are frozen; however, the increased risk of aneuploidy remains. These factors can each have significant detrimental impacts on reproductive outcomes. By adjusting for the increasing rates of adenomyosis with age, this study importantly accounts for these effects, rather than assuming that the observed reduced fertility is due to adenomyosis.

A secondary aim of the current study was to try to establish where and when SEOA might have an impact on the IVF and conception process. The unadjusted analysis points to higher cycle cancellation rates, fewer eggs collected and fewer useful embryos produced, and so seemingly results in more cycles where no embryo is transferred. Contrary to other studies, the rate of pregnancy loss was lower in the SEOA group (Chiang *et al.*, 1999; Maubon *et al.*, 2010; Youm *et al.*, 2011; Salim *et al.*, 2012; Stanekova *et al.*, 2018; Sharma *et al.*, 2019). Explanations for the loss of significance of these outcome measures with adjusting suggests an inadequate sample size, the possibility that maternal age and smoking are the true risk, or that any effect of adenomyosis occurs earlier in the process, around egg production and embryo development, rather than at the currently understood points of implantation and the first-trimester pregnancy survival.

Interestingly, the prevalence of adenomyosis in women undergoing IVF in this study is 32%, which is higher than the suggested prevalence in the general population of 8–27% (Maheshwari *et al.*, 2012; Naftalin *et al.*, 2012). This could be attributed to this study being prospective, the subspecialists performing the ultrasounds who may, therefore, be more likely to find subtle SEOA and inconsistencies in the diagnostic criteria. This study defined adenomyosis as the presence of one or more SEOA but standardised diagnostic criteria do not exist and some studies require more features before diagnosing adenomyosis. However, when we used presence of three MUSA criteria to define adenomyosis, we still did not find a significant effect on live birth rates. Finally, women undergoing IVF are not necessarily representative of the general population and the prevalence of adenomyosis may, therefore, be higher in this group with infertility (de Souza *et al.*, 1995; Zangos *et al.*, 2004; Benagiano *et al.*, 2009).

This study’s strengths lie in its large sample size and design. Of the studies examining the relation between IVF and adenomyosis, this study has the second largest population of women with SEOA, which is almost double the size of any single study included in the aforementioned meta-analyses (Vercellini *et al.*, 2014; Younes and Tulandi, 2017): it, therefore, contributes a substantial amount of data to the literature. Its prospective nature helps to further strengthen this study by reducing the potential for retrospective biases. Other strengths of our study include the following: diagnostic criteria were clearly identified at the beginning of the study, ultrasounds performed by select specialised sonographers and cases with uncertain sonographic findings were excluded to maximise the accuracy in identification of SEOA. Finally, adjusting outcomes for potentially confounding effects has added to the rigor of this study.

Despite the above strengths, there are also several limitations. First, despite a sample size calculation beforehand, this study was underpowered as no allowance was made for the potential effect of confounding factors in reducing the difference in outcomes, nor did we account for the loss of patients with cancelled or non-transfer cycles. ETs occurred in 43% of SEOA patients and 52% of controls, suggesting that the study populations should have been 220 and 660, respectively, rather than our estimated 130 and 335. The use of ultrasound alone for diagnosis of adenomyosis is a further limitation given the possibility of false positives and negatives (de Souza *et al.*, 1995; Champaneria *et al.*, 2010) and possible inter-observer variability between sonographers. Ultrasound, however, is considered highly accurate and it would not be practical to obtain histological samples in this population. The lack of a standardised diagnostic definition for SEOA and scan quality reduces inter-study reliability as variations lead to conflicting diagnoses and, hence, results. Additionally, it is unclear whether the location or extent of adenomyosis is significant and this was not investigated in this study. This is something that the authors believe needs to be addressed to allow for future research into IVF and adenomyosis.

This study was performed in the context of IVF and the results cannot be extrapolated to spontaneous fertility as the populations are different and IVF has previously been reported as having both possible benefits and negative effects on adenomyosis (Mijatovic *et al.*, 2010).

The comparison of fresh and frozen ETs has shown a higher crude live birth rate in the total frozen transfers than total fresh transfers, at 23.6% and 15.5%, respectively. The difference in predicted live birth between women with and without SEOA was a drop of 4.5% and an increase of 4.0% in fresh and frozen transfers, respectively. Separate from suggesting an overall better outcome with frozen transfers, this specifically raises the question; do women with SEOA get higher live birth rates with planned frozen transfers?

This study suggests that initial beliefs that SEOA worsens IVF outcomes may in fact be overstated, with confounding factors inflating the true effect. Larger correctly powered studies are required to clarify the impact, if any, of SEOA on IVF. In particular, it would be important to minimise the bias caused by the association between increased age and adenomyosis. This could be done by comparing the reproductive outcomes of embryos from donor oocytes or focusing only on high quality frozen ETs that have been submitted to PGT.

## Supplementary data

Supplementary data are available at *Human Reproduction Open* online

## Data availability

The data underlying this article will be shared on reasonable request to the corresponding author.

## Supplementary Material

hoab015_Supplementary_Data
