# Supplementary material for: The impact of adenomyosis on IVF outcomes: a prospective cohort study
Source: Hum Reprod Open. 2021 Apr 19;2021(2):hoab015. doi: 10.1093/hropen/hoab015 (PMC8054136; doi:10.1093/hropen/hoab015)
Supplement: hoab015_Supplementary_Data [file hoab015_supplementary_data.docx]

**Supplementary Table SI** Comparison of outcomes of initiated stimulation cycles and cycles with a fresh embryo transfer (severe SEOA versus no SEOA).

|  | | **Severe SEOA** | **No SEOA** | **Crude OR** | **p** | **Adjusted^a^ OR** | **p** |
| --- | --- | --- | --- | --- | --- | --- | --- |
| **Initiated stimulation cycles** | | | | | | | |
|  | | **N = 132** | **N = 643** |  | | | |
| **Clinical Pregnancy** | | 8.3% (11/132) | 17.3% (111/643) | 0.7 (0.5-0.9) | **0.01** | 0.5 (0.3-1.0) | 0.052 |
| **Live Birth** | | 6.8% (9/132) | 8.9% (57/643) | 0.9 (0.6-1.3) | 0.44 | 0.9 (0.4-2.0) | 0.84 |
| **Cycles with fresh ET** | | | | | | | |
|  | **N = 55** | | **N = 335** |  | | | |
| **Clinical Pregnancy** | 20.0% (11/55) | | 33.1% (111/335) | 0.7 (0.5-1.0) | 0.06 | 0.5 (0.2-1.1) | 0.07 |
| **Live Birth** | 16.4% (9/55) | | 17.0% (57/335) | 1.0 (0.7-1.4) | 0.91 | 1.1 (0.5-2.4) | 0.90 |

^a^ Logistic regression model included explanatory variables: age, smoking status, treatment cycle, aetiology status [endometriosis, ovulation defect, polycystic ovaries (PCO), polycystic ovary syndrome (PCOS)], BMI, parity status. This analysis used pooled results from multiple imputation data.

SEOA: sonographic evidence of adenomyosis, OR: odds ratio.

**Supplementary Table SII** Comparison of outcomes of initiated stimulation cycle – limited to subject’s 1^st^ IVF cycle, by presence of SEOA.

| **Result of Cycle with fresh ET** | **With SEOA (N=221)** | **Without SEOA (N=499)** | **Crude OR** | **p** | **Adjusted^a^ OR** | **p** |
| --- | --- | --- | --- | --- | --- | --- |
| **Clinical Pregnancy** | 8.6% (19/221) | 16.4% (82/499) | 0.5 (0.3-0.8) | **<0.01** | 0.6 (0.3-1.0) | **0.03** |
| **Live Birth** | 5.0% (11/221) | 9.0% (45/499) | 0.5 (0.3-1.0) | 0.07 | 0.6 (0.3-1.2) | 0.16 |

^a^ Logistic regression model included explanatory variables: age, smoking status, treatment cycle, aetiology status (endometriosis, ovulation defect, PCO, PCOS), BMI, parity status. This analysis used pooled results from multiple imputation data. Data presented as % (n).

**Supplementary Table SIII** Comparison of outcomes of initiated stimulation cycle – limited to nulliparous subjects, by presence of SEOA.

| **Result of Cycle with fresh ET** | **With SEOA (N=280)** | **Without SEOA (N=604)** | **Crude OR** | **p** | **Adjusted^a^ OR** | **p** |
| --- | --- | --- | --- | --- | --- | --- |
| **Clinical Pregnancy** | 9.6% (27/280) | 17.4% (105/604) | 0.5 (0.3-0.8) | **<0.01** | 0.6 (0.4-0.9) | **0.03** |
| **Live Birth** | 4.3% (12/280) | 8.9% (54/604) | 0.5 (0.2-0.9) | **0.02** | 0.5 (0.3-1.1) | 0.07 |

^a^ Logistic regression model included explanatory variables: age, smoking status, treatment cycle, aetiology status (endometriosis, ovulation defect, PCO, PCOS), BMI, parity status. This analysis used pooled results from multiple imputation data. Data presented as % (n).
